# Predicting changes in cataract surgery health outcomes using a cataract surgery appropriateness and prioritization instrument

**DOI:** 10.1371/journal.pone.0246104

**Published:** 2021-01-28

**Authors:** Morgan E. Lim, Simona C. Minotti, Chelsea D’Silva, Robert J. Reid, Matthew B. Schlenker, Iqbal K. Ahmed

**Affiliations:** 1 Institute for Better Health, Trillium Health Partners, Mississauga, Ontario, Canada; 2 Institute for Health Policy, Management and Evaluation, University of Toronto, Toronto, Ontario, Canada; 3 Department of Statistics and Quantitative Methods, University of Milano-Bicocca, Milano, Italy; 4 Department of Surgery, Trillium Health Partners, Mississauga, Ontario, Canada; 5 Prism Eye Institute, Oakville, Ontario, Canada; University of Warmia, POLAND

## Abstract

**Objective:**

Determine whether items in a cataract surgery appropriateness and prioritization questionnaire can predict change in best corrected visual acuity (BCVA) and health related quality of life (HRQOL) following cataract surgery.

**Methods:**

313 patients with a cataract in Ontario, Canada were recruited to participate. BCVA was measured using the Snellen chart. HRQOL was measured using a generic instrument (EQ5D), a visual functioning instrument (Catquest-9SF), and an appropriateness and prioritization instrument (17 items). Outcomes were measured preoperatively and 3–6 months postoperatively. Descriptive statistics were used to describe demographics and outcomes. For each appropriateness and prioritization questionnaire item, a one-way ANOVA was used to compare group means of the change in BCVA, EQ5D, and Catquest-9SF.

**Results:**

Participants had a mean age of 69 years and were 56% female. BCVA improved in 81%, EQ5D in 49.6%, and Catquest-9SF score in 84% of patients. Improvement in both BCVA and Catquest-9SF scores were found in 68.5% of patients. The ANOVA showed a statistically significant association between a change in BCVA and the ability to participate in social life, and a statistically significant association between a change in Catquest-9SF and glare, extent of impairment in visual function, safety and injury concerns, ability to work and care for dependents, ability to take care of local errands, ability to assist others and ability to participate in social life.

**Conclusions:**

Almost all patients had improved BCVA and/or visual functioning after surgery. Seven variables from the cataract appropriateness and prioritization instrument were found to be predictors of improvement in Catquest-9SF measuring visual functioning.

## Introduction

Cataract remains the leading cause of blindness and vision loss in the world. Visual impairment impacts an individual’s quality of life by hindering their ability to perform daily living activities, to work and to care for dependents [1]. Reduced vision can also lead to an increase in fall-related injuries and fractures where 20% can result in hospitalizations [2]. These deleterious effects will differ from patient to patient depending on the severity of the cataract.

Cataract blindness is rare in economically developed countries because there is greater access to cataract surgery. The demand for cataract surgery, however, has increased dramatically due to the rising prevalence of cataracts in the aging population, so that, across many countries in the developed world, surgical capacity will likely not grow fast enough to keep up with this growing demand [3]. For instance, in Ontario Canada, projections for cataract demand will increase between 72%-144% in the next 25 years [3, 4] but there will be substantially slower growth of publically-funded outpatient operative capacity.

In an attempt to address this concern a number of appropriateness and prioritization instruments for cataract surgery have been developed but with varied success [5, 6]. This can be attributed to low predictive validity (e.g. inadequate sample size, and missing confounders) or poor implementation. More importantly, the literature has demonstrated weak to no association of these instruments with clinical and visual functioning outcomes that they were supposed to predict [7]. For example, preoperative best corrected visual acuity (BCVA) has been proposed as a measure of appropriateness, but the literature is inconsistent on whether it is a good predictor of postoperative BCVA [7].

In an effort to incorporate patient oriented measures, a cataract appropriateness and prioritization instrument was modified in Ontario, Canada to reflect the local context [8]. Considering the evidence in the literature of a counter intuitive association between appropriateness and prioritization classification and health outcomes, it is important to understand the ability of the instrument to predict change in general and vision-related health outcomes. Understanding these predictors may help clinicians assess patients that are most likely to benefit from surgery, giving higher priority to more highly impacted patients [7, 9]. The purpose of this study is to determine whether items in the modified cataract surgery appropriateness and prioritization instrument could predict change in BCVA, visual functioning and health related quality of life (HRQOL) following cataract surgery.

## Methods

### Setting, study design, recruitment

This study was conducted in the Region of Peel in Ontario, one of the most ethnically, culturally, and socio-economically diverse areas of Canada. It is home to 1.38 million citizens with 51% of residents born outside of Canada and over 18,000 newcomers from across the world arriving each year. A multi-centre prospective cohort study design was used. Consecutive sampling was used to recruit patients prior to their cataract surgery patients from two ophthalmologic clinics. At their consultation appointment, eligible patients were invited to participate in the study and written consent was obtained. Patients missed in clinic were recruited over the phone and then mailed a written consent form to sign and return. The inclusion criteria is presented in Table 1. The Trillium Health Partners Research Ethics Board (ID#777) approved this study which adheres to the guidelines in the Declaration of Helsinki.

**Table 1:** Participant inclusion criteria.

Inclusion Criteria
1) Aged 40–85
2) Diagnosed with a cataract
3) Scheduled for cataract surgery before May 2018
4) Ability to speak, read and write in English (or have a family member present to assist)
5) Clinically competent as assessed by the Short Orientation-Memory-Concentration Test [10]
6) Not experiencing unmanageable chronic pain
7) Scheduled for unilateral cataract surgery
8) Completed both ultrasound and laser biometry IOL testing prior to surgery
9) Implanting a monofocal lens
10) ≥ 4 weeks between surgeries on contralateral eye
11) ≥ 4 months since any other eye surgery.

### Measurement

#### Cataract surgery appropriateness and prioritization questionnaire

**Fig 1:**
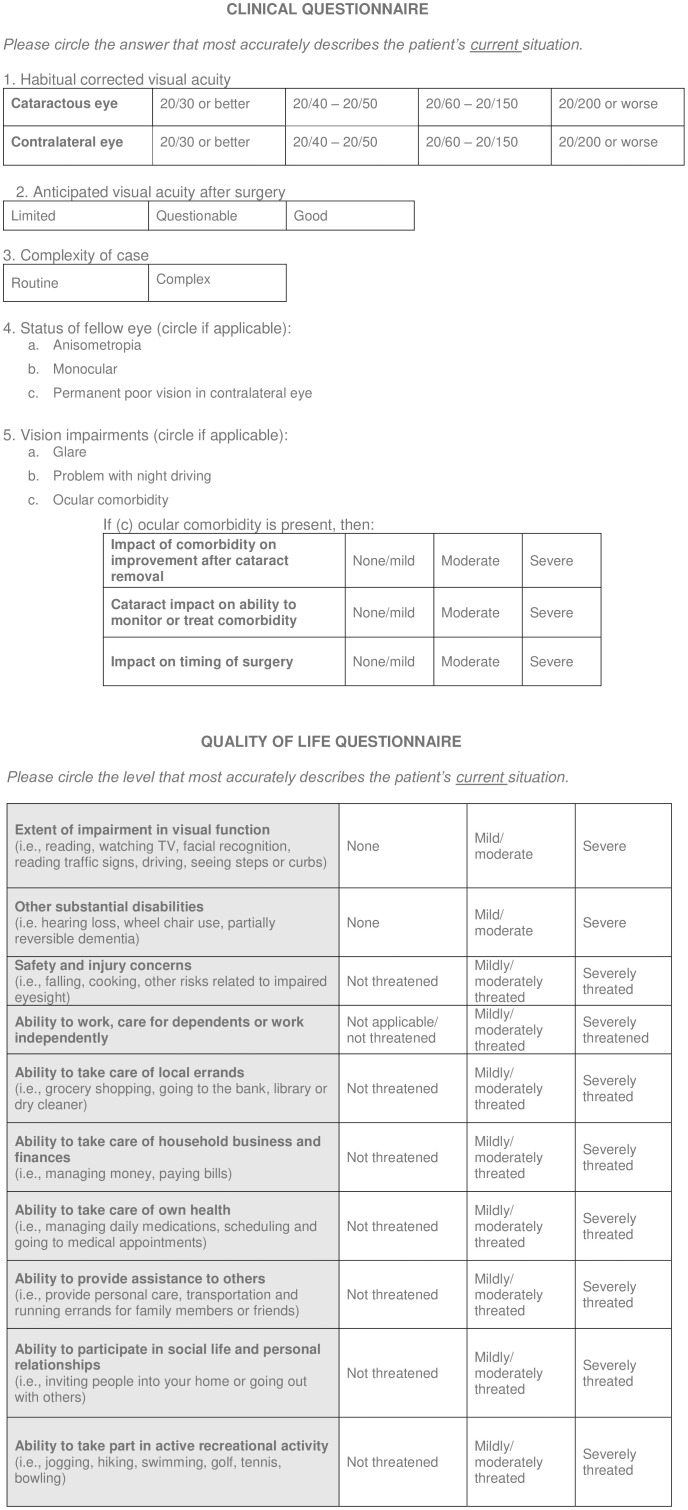
Cataract surgery prioritization instrument.

The Western Canada Wait List Project (WCWLP) cataract surgery prioritization instrument was modified [8] as follows (Fig 1): 1) revision of criteria to assess alignment with current clinical practice, 2) selection of criteria based on gaps and redundancies in the WCWLP measurement instrument, 3) a modified Delphi process with an expert panel to select criteria to include for evaluating appropriateness and priority for surgery and 4) a reliability study using a G-theory framework [11].

The first section includes a clinical questionnaire completed by the ophthalmologist to assess the BCVA, complexity of the case, anticipated VA after surgery, status of the contralateral eye, and whether the patient struggles with glare, night driving or other ocular comorbidities. The second section incorporates a self-administered HRQOL questionnaire where patients assess the extent of impairment in their visual function, presence of any other disabilities, safety concerns, and impact on their ability to work or care for dependents, take care of errands, take care of household business, take care of their own health, provide assistance to others, participate in a social life, and take part in recreational activities.

#### Health outcomes

Clinical and self-reported HRQOL outcomes were measured to assess cataract surgery.

BCVA is a clinical measure of the distance at which the smallest object can be visually processed and will be measured using the Snellen chart [12]. The chart is printed with 11 lines of block letters where the top line has the largest letters and letters on subsequent rows get progressively smaller. The line that is legible to the patient indicates their VA and is converted to a linear scale known as logMAR units [12]. It is best corrected when the patient is measured wearing glasses or contact lens correction. Patients can also be categorized into the following: 20/30 or better, 20/40 to 20/50, 20/60 to 20/150 and 20/200 or worse.

The EQ5D questionnaire is a standardized instrument for measuring general health status [13]. It is a self-administered questionnaire that measures 5 dimensions of health: mobility, self-care, usual activities, pain/discomfort and anxiety/depression. The responses from the EQ5D questionnaire can be converted into an overall health utility index ranging from 0.0 (death) to 1.0 (perfect health) through the Canadian algorithm [13].

Visual functioning describes how well the patient functions in vision-related daily activities. It can be measured using the Catquest-9SF questionnaire [14]. The 9 item self-administered questionnaire [14, 15] consists of two global items and seven difficulty items measured on a 4-point Likert scale (0 = very great difficulty, 1 = great difficulty, 2 = some difficulty, 3 = no difficulty). The responses can be recoded using Rasch analysis so that parametric statistics can be applied. Rasch analysis expresses responses in logit units, which is the natural log odds of a participant having complete difficulty versus no difficulty [16]. The Catquest 9SF questionnaire has been validated in Canadian, Swedish and Australian populations [17, 18] and has shown the greatest responsiveness in a head-to-head comparison with 16 other cataract surgery outcome questionnaires [16, 17].

### Data collection

Data were collected preoperatively and 3 to 6 months postoperatively. The following data were collected preoperatively: demographic data (age, sex, marital status, ethnicity, education level, household income, and non-ocular comorbidities), the modified cataract surgery appropriateness and prioritization questionnaire and health outcomes. The Research Associate (RA) administered a questionnaire package that included the cataract appropriateness and prioritization questionnaire, the EQ5D and the Catquest-9SF. An RA abstracted clinical data from medical charts and completed a paper case report form with patients for the remainder of questions.

Approximately 3 to 6 months after surgery, participants were phone called to complete the post-surgery questionnaire composed of the Catquest-9SF and EQ5D. Physical copies of the post-surgery questionnaire were mailed to participants if unable to complete over the phone.

Postoperative BCVA was collected from multiple sources as not all cataract patients return to their ophthalmologist after surgery and very few optometrists return BCVA to the patients following surgery. First, if participants indicated that they had seen their optometrist, BCVA measures were requested from the optometrist offices. The second method for collecting post-operative BCVA was through chart abstraction. The BCVA of patients who did not indicate seeing their optometrist or whose optometrist did not return their BCVA was abstracted from their charts ranging from 1 week to 6 month post-surgery.

### Statistical analysis

Descriptive statistics were used to describe baseline characteristics and outcomes through frequency tables, means with standard deviations and medians with inter-quartile ranges as appropriate. Histograms and scatterplots were also used to describe the data. Effectiveness of cataract surgery was calculated as the mean change (postoperative minus preoperative) in BCVA, EQ5D, and Catquest-9SF scores. To assess the predictive ability of each appropriateness and prioritization questionnaire item, a one-way Anova was used to compare the means of each outcome (BCVA and Catquest-9SF) across groups defined by the possible answers to the item [19]. A one-way ANOVA was not used to compare the means for EQ5D because the normality assumption for the standardized residuals was violated. Statistical significance was set at 0.05. All analyses were conducted using IBM SPSS Statistics 25 [20].

## Results

**Fig 2:**
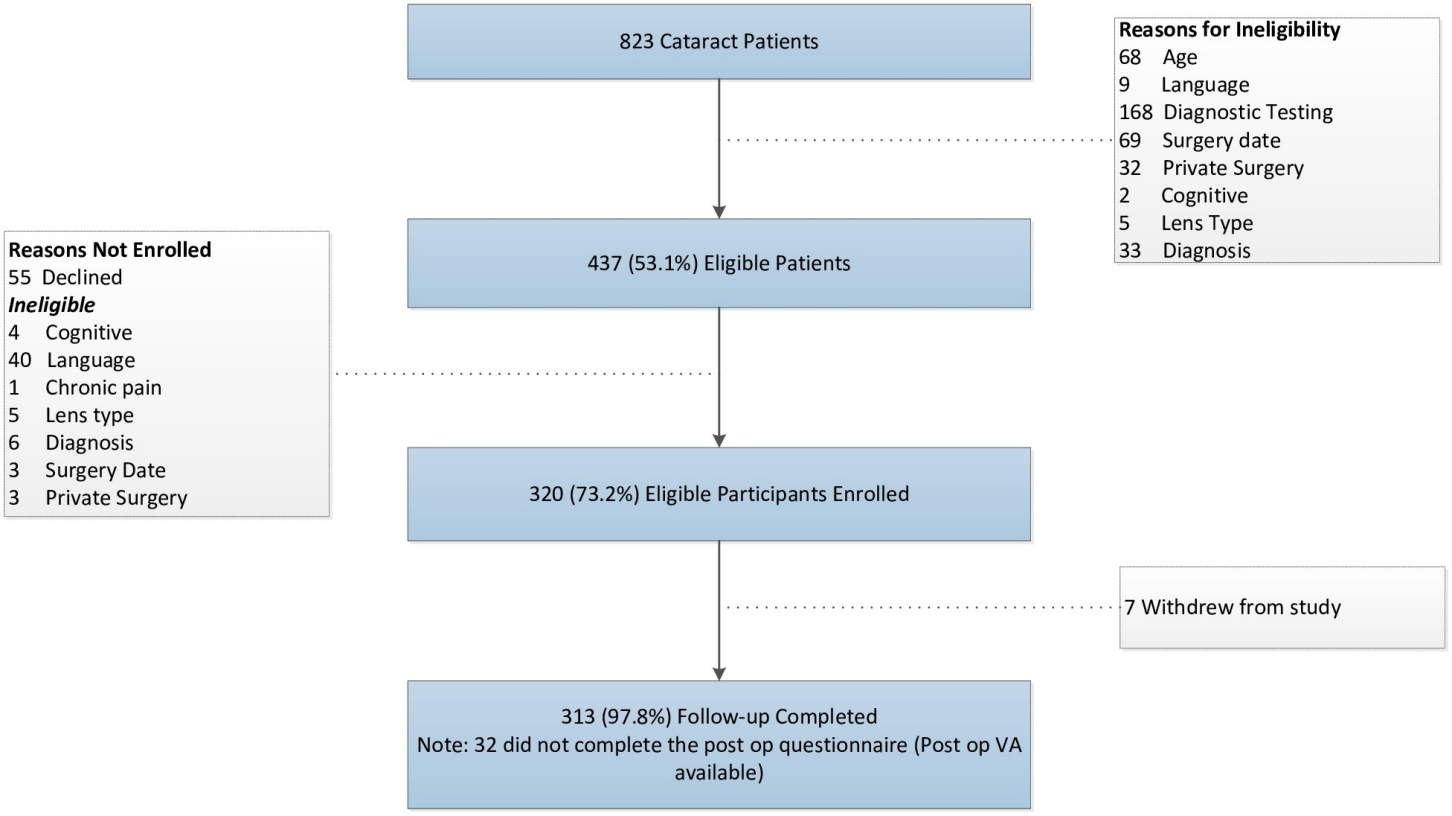
Patient recruitment.

A total of 320 eligible participants completed the preoperative data collection, and 7/320 (2.0%) withdrew from the study (Fig 2). Postoperative BCVA was collected for 312/313 (99.7%): half were retrieved from optometrists and half were abstracted from the ophthalmologist’s charts. Postoperatively, 281/313 (89.7%) participants completed the follow-up questionnaires for EQ5D and Catquest-9SF. The average age of participants was 69 years and 56.5% were female (Table 2). The distribution of household income varied across categories. All participants had an education level of high school or above, 31.9% graduated from university.

**Table 2:** Participant demographics.

Demographics	Percentage n = 313
Average age (y)	69.07 (42–84)
Sex (% female)	56.5%
Ethnicity (%)	
Africa	3.9%
Americas	27.7%
Asia	28%
Europe	40.5%
Household Income (%)	
<$30000	23.4%
$30000-$49999	25.5%
$50000-$69999	16.1%
$70000 +	34.9%
Education (%)	
LT High School	15.3%
High school	26.8%
Apprenticeship	5.1%
College	20.8%
University	31.9%

Preoperative cataractous eye BCVA was evenly distributed between categories whereas preoperative contralateral eye BCVA was more skewed towards good BCVA categories (20/30 or better and 20/40 to 20/50). Surgical cases were predominantly routine (89%) with good anticipated BCVA postoperatively (88.5%). Most patients did not have anisometropia (98%), monocularity, or permanent poor vision in the contralateral eye. Two thirds of patients experienced glare, 56.9% reported problems with night driving and 36.7% had ocular comorbidities. About 70% of patients reported no issues in HRQOL except for items related to extent of impairment in visual function (none– 25.6%, mild/moderate– 56.5%, severe– 17.6%).

### Change in health outcomes

#### BCVA

**Fig 3:**
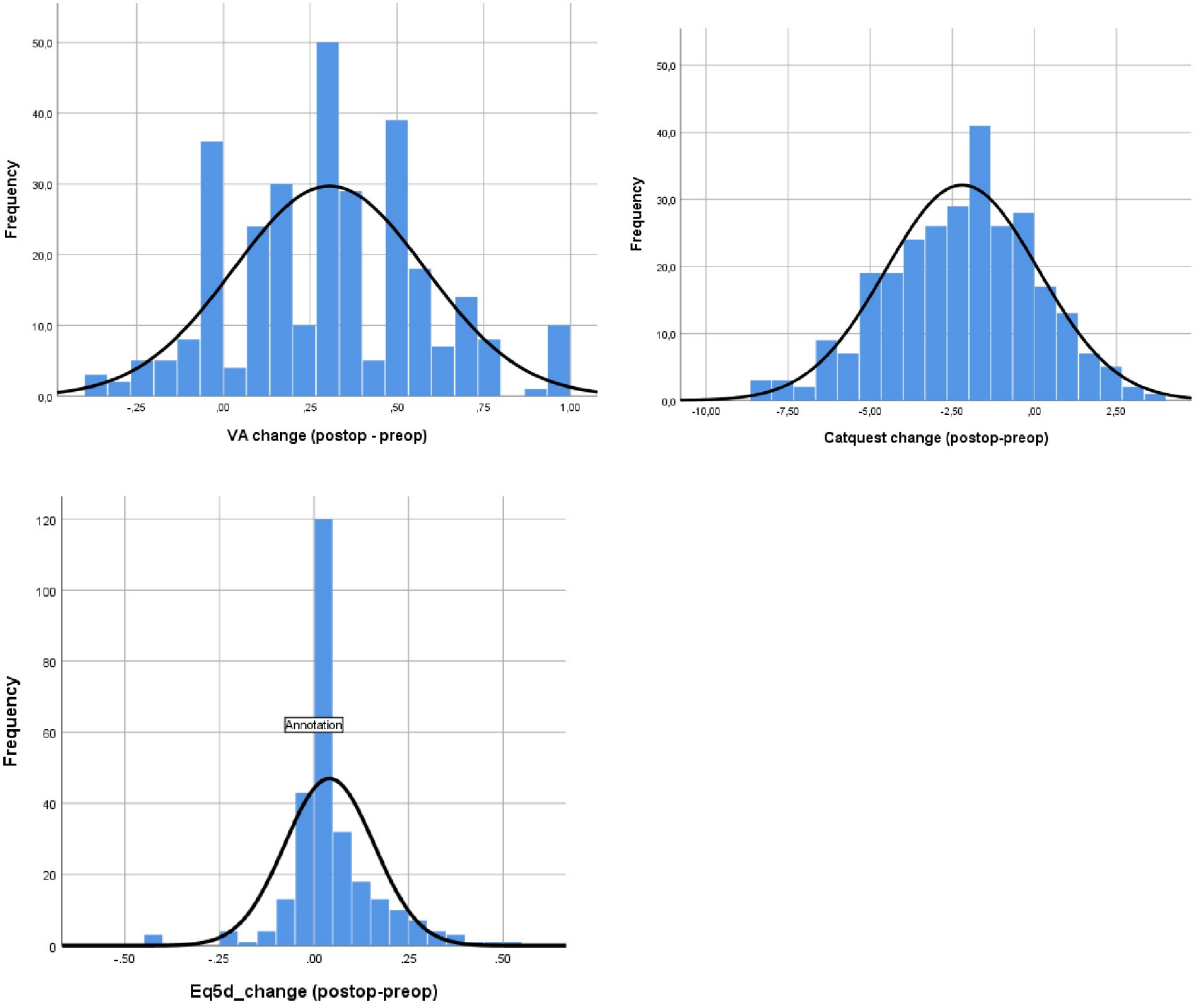
Histograms of change in best corrected visual acuity and change in Catquest-9SF.

The majority of participants (80.8%) experienced improvements in BCVA after having cataract surgery (Table 3). Those with a good preoperative BCVA (20/30 or better) were less likely to see improvements in BCVA following surgery. Of the patients with good vision, 27% had no change in BCVA postoperatively and 20% worsened. Additionally, 82% of patients without comorbidities improved versus 77% of patients who did have a comorbidity. Fig 3 describes the distribution of change in BCVA which does not fit a normal distribution.

**Table 3:** Change in best corrected visual acuity and Catquest-9SF following cataract surgery.

Preoperative	N	Change in Outcome		
		Decline	No Change	Improved
**BCVA**				
20/30 or better	60	12 (20%)	16 (26.7%)	32 (53.3%)
20/40-20/50	103	7 (6.8%)	12 (11.7%)	84 (81.5%)
20/60-20/150	83	4 (4.8%)	2 (2.4%)	77 (92.8%)
20/200 or worse	62	1 (1.6%)	5 (8.1%)	56 (90.3%)
Total	308	24 (7.8%)	35 (11.4%)	249 (80.8%)
**Catquest-9SF Score**				
9–18 (very great to great difficulty)	185	38 (20.5%)	0 (0%)	147 (79.5%)
19–27 (great to some difficulty)	83	6 (7.2%)	0 (0%)	77 (92.8%)
28–36 (some to no difficulty)	13	1 (7.7%)	0 (0%)	12 (92.3%)
Total	281	45 (16.0%)	0 (0%)	236 (84.0%)

#### Catquest-9SF

The preoperative mean Catquest-9SF score was -1.43 (SD = 1.68) and postoperative was -3.7 (SD = 1.99); more negative indicates an improvement in visual function (Fig 3). Table 3 describes the change in Catquest-9SF by preoperative Likert score. The Catquest-9SF Likert score was categorized into three groups (9–18 = very great to great difficulty, 19–27 = great to some difficulty, 28–36 = some to no difficulty on all items) to provide further context. Of those that completed the pre and post cataract surgery Catquest-9SF, 84% experienced an improvement in Catquest-9SF score.

#### EQ5D

Overall, 49.6% improved, 25.9% had no change and 24.5% worsened. The preoperative mean EQ5D score was 0.85 (SD = 0.14) and the postoperative mean EQ5D score was 0.88 (SD = 0.12) with an average change of 0.04.

### Association between health outcomes

The proportion of patients with improved BCVA is similar across preoperative Catquest-9SF scores (Table 4A); however, of those patients who reported some to no difficulty (Likert scores 28–36) in visual functioning, 94% had improved BCVA postoperatively. Similarly, the proportion of patients with improved Catquest-9SF were similarly distributed across preoperative BCVA; however, patients with good preoperative BCVA (15%) still reported worse Catquest-9SF postoperatively (Table 4B). Patients with improvement in EQ5D were more likely to have improvement in BCVA and Catquest-9SF as well (Table 4C).

**Table 4:** A. Change in best corrected visual acuity following cataract surgery by Catquest-9SF scores. B. Change in Catquest-9SF scores following cataract surgery by best corrected visual acuity. C. Change in best corrected visual acuity following cataract surgery by change in EQ-5D.

**A**				
**Preoperative**	**N**	**Change in BCVA**		
		**Decline**	**No Change**	**Improved**
**Catquest-9SF Score**				
9–18 (very great to great difficulty)	201	18 (9.0%)	28 (13.9%)	155 (77.1%)
19–27 (great to some difficulty)	91	5 (5.5%)	7 (7.7%)	79 (86.8%)
28–36 (some to no difficulty)	16	1 (6.3%)	0 (0%)	15 (93.8%)
Total	308	24 (7.8%)	35 (11.4%)	249 (80.8%)
**B**				
**Preoperative**	**N**	**Change in Catquest-9SF**		
		**Decline**	**No Change**	**Improved**
**BCVA**				
20/30 or better	58	9 (15.5%)	0 (0%)	49 (84.5%)
20/40-20/50	93	14 (15.1%)	0 (0%)	79 (84.9%)
20/60-20/150	76	16 (21.1%)	0 (0%)	60 (78.9%)
20/200 or worse	54	6 (11.1%)	0 (0%)	48 (88.9%)
Total	308	45 (16.0%)	0 (0%)	236 (84%)
**C**				
	**N**	**Change in BCVA**		
		**Decline**	**No Change**	**Improved**
**EQ5D change > 0**	137	11 (8.0%)	11 (8.0%)	115 (83.9%)
	**N**	**Change in Catquest-9SF**		
		**Decline**	**No Change**	**Improved**
**EQ5D change > 0**	138	18 (13.0%)	0 (0%)	120 (87.0%)

**Fig 4:**
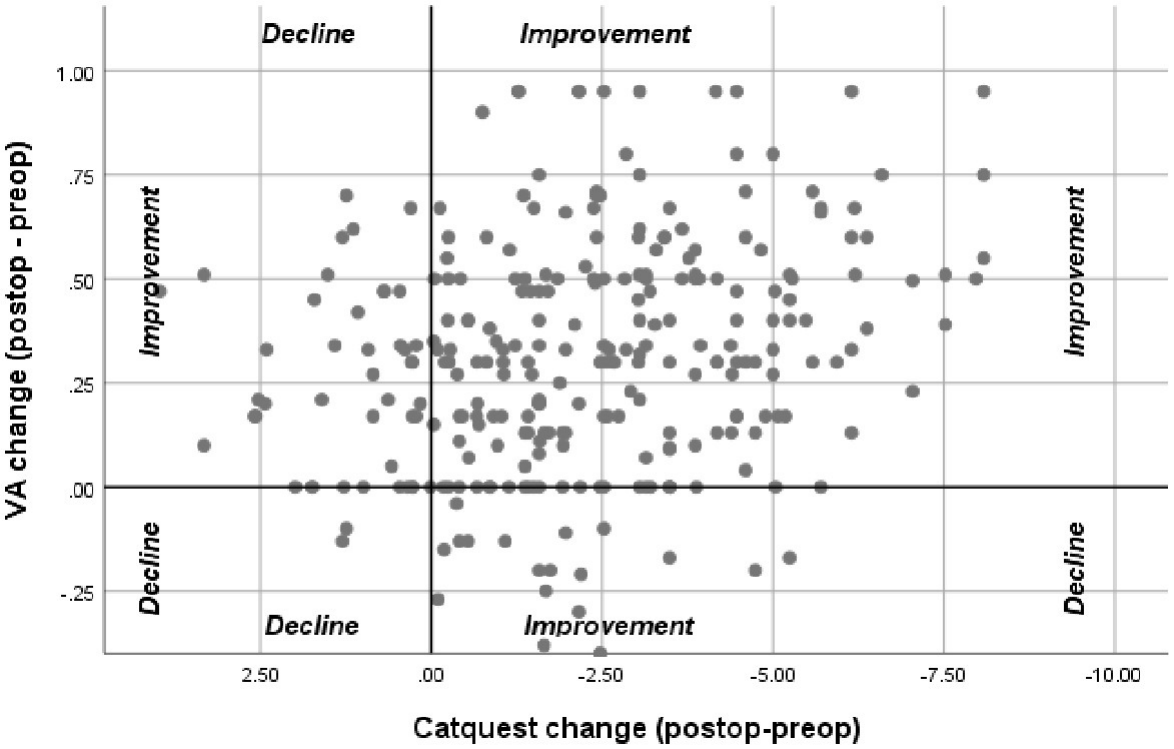
Scatterplot of change in best corrected visual acuity versus change in Catquest-9SF.

Fig 4 shows minimal association between change in BCVA and change in Catquest-9SF. The majority of patients (61%) had improvement in BCVA and Catquest-9SF. For these patients, 71% experienced glare and 58% had problems with night driving.

### Predicting change in health outcomes

In Table 5, the one-way ANOVA showed a statistically significant association between a change in BCVA and the ability to participate in social life. It also showed a statistically significant association between a change in Catquest-9SF and the following items: glare, extent of impairment in visual function, safety and injury concerns, ability to work and care for dependents, ability to take care of local errands, ability to assist others and ability to participate in social life. The same significant associations between criteria items and change in Catquest-9SF and BCVA were found when adjusted for age and gender.

**Table 5:** ANOVA results for cataract surgery appropriateness and prioritization items.

Variable	Categories		Change in Catquest-9SF				Change in BCVA			
			Mean	N	F	Sig.	Mean	N	F	Sig.
Preop BCVA of eye for surgery- categories	20/200 or worse		-2.71	54	1.41	0.24				
	20/60-20/150		-2.10	76						
	20/40-20/50		-2.23	93						
	20/30 or better		-1.83	58						
Preop BCVA of contralateral eye- categories	20/200 or worse		-1.75	14	0.59	0.62				
	20/60-20/150		-1.95	37						
	20/40-20/50		-2.15	106						
	20/30 or better		-2.38	124						
Anticipated BCVA after surgery	Limited		-1.42	5	1.03	0.36				
	Questionable		-1.64	22						
	Good		-2.27	252						
Anisometropia	No		-2.19	276	0.27	0.61	0.30	302	0.17	0.68
	Yes		-2.73	5			0.35	6		
Monocular	No		-2.20	278	0.02	0.89	0.31	305	1.48	0.23
	Yes		-2.38	3			0.11	3		
Permanent poor vision in fellow eye	No		-2.23	277	3.28	0.07	0.31	303	0.86	0.35
	Yes		-0.12	4			0.19	5		
Complexity of case	Routine		-2.27	251	2.00	0.16	0.30	274	0.07	0.79
	Complex		-1.58	25			0.32	29		
Does the patient experience glare	No		-1.65	86	7.33	**0.01**	0.28	97	1.06	0.31
	Yes		-2.46	194			0.32	210		
Does the patient have problem with night driving	No		-1.94	120	2.99	0.09	0.31	132	0.01	0.93
	Yes		-2.42	160			0.30	175		
Is ocular comorbidity present	No		-2.23	175	0.06	0.81	0.31	194	0.39	0.53
	Yes		-2.16	105			0.29	113		
Extent of impairment in visual function	None		-1.55	72	7.17	**0.00**	0.26	79	2.50	0.08
	Mild/Moderate		-2.25	160			0.31	173		
	Severe		-3.14	48			0.36	55		
Other substantial disabilities	None		-2.26	229	0.22	0.80	0.30	253	0.11	0.90
	Mild/Moderate		-2.06	44			0.31	47		
	Severe		-2.62	6			0.26	6		
Safety and injury concerns	None		-2.10	205	4.55	**0.01**	0.31	221	1.32	0.27
	Mild/Moderate		-2.16	57			0.26	65		
	Severe		-3.79	18			0.36	21		
Ability to work, care for dependents	None		-2.03	228	5.36	**0.01**	0.30	248	0.36	0.70
	Mild/Moderate		-2.86	40			0.30	43		
	Severe		-3.98	10			0.37	13		
Ability to take care of local errands	None		-1.96	219	7.25	**0.00**	0.30	239	0.22	0.80
	Mild/Moderate		-3.00	50			0.32	56		
	Severe		-3.85	11			0.35	12		
Ability to take care of household business	None		-2.16	254	0.89	0.41	0.31	278	0.16	0.86
	Mild/Moderate		-2.78	24			0.28	26		
	Severe		-3.00	2			0.26	3		
Taking care of your own health	None		-2.15	261	2.41	0.09	0.31	286	0.36	0.70
	Mild/Moderate		-2.95	16			0.25	18		
	Severe		-4.51	3			0.32	3		
Ability to provide assistance to others	None		-2.07	248	4.61	**0.01**	0.31	271	0.15	0.87
	Mild/Moderate		-3.41	26			0.33	28		
	Severe		-3.22	6			0.27	8		
Ability to participate in social life	None		-1.99	238	10.73	**0.00**	0.31	259	3.70	**0.03**
	Mild/Moderate		-3.17	32			0.24	37		
	Severe		-4.92	9			0.51	10		
Take part inactive recreational activities	None		-2.14	212	0.71	0.49	0.31	235	0.53	0.59
	Mild/Moderate		-2.55	47			0.27	49		
	Severe		-2.45	20			0.32	22		

## Discussion

Internationally, health systems continue to struggle with how to allocate and prioritize resources for elective surgeries. It is a system issue involving transformation of process and funding. In Canada, population growth is forcing governments to make funding reforms to the public healthcare system. Further emphasis has been placed on delisting procedures and appropriateness of treatments and diagnostics. The purpose of this study was to assess the ability of the items in a modified cataract surgery appropriateness and prioritization instrument [8] to predict three outcomes (BCVA, visual functioning, HRQOL).

This study identified seven statistically significant predictors of an improvement in the Catquest-9SF score: glare, extent of impairment in visual function, safety and injury concerns, ability to work and care for dependents, ability to take care of local errands, ability to assist others and ability to participate in social life. This study also found a statistically significant association between an improvement in BCVA and the ability to participate in social life. This is contrary to other studies using appropriateness tools whereby patients rated as inappropriate or uncertain were more likely to have no change or a decline in BCVA than those rated as appropriate [21–23]. Another study found that high priority patients experienced greater improvement in BCVA [24]. In our population, the lack of association with a change in BCVA could be due to low variation in patients’ health outcomes or in questionnaire administration. BCVA is taken monocularly, whereas functioning is based on use of both eyes functioning together. BCVA is likely more sensitive to significant monocular limitation, though does not always capture the visual disability that a bilateral cataract process can cause. BCVA also may not encompass visual disability because patients can have good high contrast BCVA, but still suffer from other visual limitations such as glare. The issue becomes less about clinical measures and more about every day functioning. Based on this principle, the predictors found to be associated with an improvement in Catquest-9SF should be further explored as potentially useful to prioritize patients.

This study also showed that an improvement in BCVA did not correlate with an improvement in Catquest-9SF. This is counterintuitive to how other cataract surgery appropriateness instruments are designed to categorize patients [25]. Fig 4 shows no pattern in BCVA change for those patients who reported good Catquest-SF scores preoperatively and postoperatively. The literature also supports that a change in BCVA is not strongly associated with change in visual functioning [15, 23, 26–28]. There continues to be a need for improvement to be measured using both clinical and patient-report outcomes; however, this creates greater difficulty in developing an appropriateness or prioritization instrument which is multi-factorial. We recommend further research into these specific items when considering appropriateness and prioritization.

Despite this study being based on a large prospectively collected sample there are some limitations. Other statistical analyses were considered, such as CHAID and CART, but the study was not designed for this purpose and therefore not powered for these analyses. Unfortunately in Canada we do not have registries used in ophthalmological practice that contain sufficient item and outcome information. Further research is needed to understand patients without improvement in outcomes postoperatively. In addition, data were not collected on whether this was the patient’s first or second eye surgery which may impact the findings, as research shows that patients undergoing second eye surgery are likely to show a smaller improvement in visual functioning since they report a higher baseline score [29]. Nevertheless, there are some strengths as it was a multi-clinic study and is one of the first studies to compare preoperative BCVA and preoperative Catquest-9SF.

Decisions about the appropriateness and prioritization of cataract surgery are complex [30]. They cannot be solved with a questionnaire and governments and healthcare providers need to look towards reforming the process. For instance, standardized referral processes could decrease the number of poor quality and inappropriate referrals that increase the queue for patients with cataracts. Currently, referral content lacks sufficient detail to apply any developed appropriateness or prioritization instrument. These instruments need to be developed in the context of the system.

## Supporting information

S1 Dataset (SAV)

## References

[1] Freeman EE, Gresset J, Djafari F, Aubin M-J, Couture S, Bruen R, et al Cataract-related vision loss and depression in a cohort of patients awaiting cataract surgery. Canadian Journal of Ophthalmology. 2009;44(2):171–6. 10.3129/i09-001 19491951

[2] Ontario Ministry of Health and Long-Term Care. Initial Report on Public Health. 2009.

[3] Hatch WV, Campbell EdL, Bell CM, El-Defrawy SR, Campbell RJ. Projecting the growth of cataract surgery during the next 25 years. Archives of Ophthalmology. 2012;130(11):1479–81. 10.1001/archophthalmol.2012.838 23143457

[4] Wang W, Yan W, Fotis K, Prasad NM, Lansingh VC, Taylor HR, et al Cataract surgical rate and socioeconomics: a global study. Investigative ophthalmology & visual science. 2016;57(14):5872–81. 10.1167/iovs.16-19894 27802517

[5] Bellan L MM. The Manitoba Cataract Waiting List Program. Canadian Medical Association Journal. 2001;164(8):1177–80. 11338806 PMC80977

[6] Conner-Spady BL SS, Courtright P, Mildon D, McGurran JJ, Noseworthy TW. The prioritization of patients on waiting lists for cataract surgery: validation of the Western Canada waiting list project cataract priority criteria tool. Ophthalmic Epidemiology. 2005;12(2):81–90. 10.1080/09286580590932770 16019691

[7] Kessel L, Andresen J, Erngaard D, Flesner P, Tendal B, Hjortdal J. Indication for cataract surgery. Do we have evidence of who will benefit from surgery? A systematic review and meta‐analysis. Acta ophthalmologica. 2016;94(1):10–20. 10.1111/aos.12758 26036605 PMC4744664

[8] Lim M, Thompson B, D’Silva C, Wang GY, Bhatnagar P, Palaganas M, et al Development and Reliability of an Appropriateness and Prioritization Instrument for Eye Care Practice: A Modified Delphi Process. Ophthalmic epidemiology. 2019:1–10. 10.1080/09286586.2019.1678653 31658845

[9] Do VQ, McCluskey P, Palagyi A, White A, Stapleton FJ, Carnt N, et al Patient perspectives of cataract surgery: protocol and baseline findings of a cohort study. Clinical and Experimental Optometry. 2018;101(6):732–9. 10.1111/cxo.12686 29675867

[10] Katzman R, Brown T, Fuld P, Peck A, Schechter R, Schimmel H. Validation of a short Orientation-Memory-Concentration Test of cognitive impairment. The American journal of psychiatry. 1983; 140(6), 734–739. 10.1176/ajp.140.6.734 6846631

[11] Streiner DL, Norman GR, Cairney J. Health measurement scales: a practical guide to their development and use: Oxford University Press, USA; 2015.

[12] Crossland M. Acuity In: Besharse J, B D, editor. The Retina and Its Disorders. 1 ed: Academic Press; 2011 p. 910.

[13] The EuroQol Group. EuroQol-a new facility for the measurement of health-related quality of life. The Journal of Health Policy. 1990;16(3):199–208. 10.1016/0168-8510(90)90421-9 10109801

[14] Lundström M, Pesudovs K. Catquest-9SF patient outcomes questionnaire: nine-item short-form Rasch-scaled revision of the Catquest questionnaire. Journal of Cataract Refractive Surgery. 2009;35(3):504–13. 10.1016/j.jcrs.2008.11.038 19251145

[15] Lundström M, Stenevi U, Thorburn W, Roos P. Catquest questionnaire for use in cataract surgery care: assessment of surgical outcomes. Journal of Cataract Refractive Surgery. 1998;24(7):968–74. 10.1016/s0886-3350(98)80053-9 9682120

[16] Ronbeck M, Lundstrom M, Kugelberg M. Study of possible predictors associated with self-assessed visual function after cataract surgery. Ophthalmology. 2011;118(9):1732–8. 10.1016/j.ophtha.2011.04.013 21715013

[17] McAlinden C, Gothwal VK, Khadka J, Wright TA, Lamoureux EL, Pesudovs K. A head-to-head comparison of 16 cataract surgery outcome questionnaires. Ophthalmology. 2011;118(12):2374–81. 10.1016/j.ophtha.2011.06.008 21945088

[18] Schlenker MB, Minotti SC, Kabanovski A, Lim M, D’Silva C, MA J, et al Catquest-9SF questionnaire and eCAPS: Validation in a Canadian Population. PLoS ONE. 2020;15(9): e0237788 10.1371/journal.pone.0237788 32976522 PMC7518613

[19] Kim H-Y. Analysis of variance (ANOVA) comparing means of more than two groups. Restorative dentistry & endodontics. 2014;39(1):74–7. 10.5395/rde.2014.39.1.74 24516834 PMC3916511

[20] IBM Corporation. IBM SPSS Statistics for Windows Version 25.0. Version 25.0 ed. Armonk, NYReleased 2017.

[21] Choi YJ, Park E-C. Analysis of rating appropriateness and patient outcomes in cataract surgery. Yonsei medical journal. 2009;50(3):368–74. 10.3349/ymj.2009.50.3.368 19568598 PMC2703759

[22] Tobacman JK, Zimmerman B, Lee P, Hilborne L, Kolder H, Brook RH. Visual acuity following cataract surgeries in relation to preoperative appropriateness ratings. Medical decision making. 2003;23(2):122–30. 10.1177/0272989X03251241 12693874

[23] Chaudhary V, Popovic M, Holmes J, Robinson T, Mak M, Eino D, et al Predictors of functional vision changes after cataract surgery: the PROVISION study. Canadian Journal of Ophthalmology. 2016;51(4):265–70. 10.1016/j.jcjo.2016.02.010 27521665

[24] Garcia‐Gutierrez S, Quintana JM, Aguire U, Barrio I, Hayas CL, Gonzalez N, et al Impact of clinical and patient‐reported outcomes on patient satisfaction with cataract extraction. Health expectations. 2014;17(6):765–75. 10.1111/j.1369-7625.2012.00801.x 22784407 PMC5060923

[25] Llorente C, Blasco JA, Quintana JM, Bilbao A, Alberdi T, Lacalle JR, et al Interhospital variation in appropriateness of cataract surgery. Journal of evaluation in clinical practice. 2011;17(1):188–95. 10.1111/j.1365-2753.2010.01421.x 20846279

[26] Charalampidou S, Loughman J, Nolan J, Stack J, Cassidy L, Pesudovs K, et al Prognostic indicators and outcome measures for surgical removal of symptomatic nonadvanced cataract. Archives of ophthalmology. 2011;129(9):1155–61. 10.1001/archophthalmol.2011.111 21555598

[27] Fong CS-u, Mitchell P, Rochtchina E, Teber ET, Hong T, Wang JJ. Correction of visual impairment by cataract surgery and improved survival in older persons: the Blue Mountains Eye Study cohort. Ophthalmology. 2013;120(9):1720–7. 10.1016/j.ophtha.2013.02.009 23664468

[28] Javed U, McVeigh K, Scott NW, Azuara-Blanco A. Cataract extraction and patient vision-related quality of life: a cohort study. Eye. 2015;29(7):921 10.1038/eye.2015.70 25976642 PMC4506347

[29] Shekhawat NS, Stock MV, Baze EF, Daly MK, Vollman DE, Lawrence MG, et al Impact of first eye versus second eye cataract surgery on visual function and quality of life. Ophthalmology. 2017;124(10):1496–503. 10.1016/j.ophtha.2017.04.014 28526550

[30] Quintana JM, Garcia S, Bilbao A, Navarro G, Perea E, de Larrea NF, et al Waiting time for cataract extraction: Predictive factors and influence on outcomes. Journal of Cataract & Refractive Surgery. 2011;37(1):19–26. 10.1016/j.jcrs.2010.07.020 21067891

